# A laboratory data-based evaluation of the efficacy and safety of generic pravastatin sodium for long-term use

**DOI:** 10.1186/s40780-015-0033-4

**Published:** 2015-12-18

**Authors:** Masanori Suzuki, Motoyasu Kanamori, Tomoaki Hashimoto, Yuji Hashimoto, Ryohkan Funakoshi, Tadanori Sasaki

**Affiliations:** Department of Pharmacy, Kameda General Hospital, 929 Higashi-cho, Kamogawa-City, 296-8602 Chiba Japan; Postgraduate Education Center, Kameda General Hospital, 929 Higashi-cho, Kamogawa-City, 296-8602 Chiba Japan; Department of Cardiology, Kameda General Hospital, 929 Higashi-cho, Kamogawa-City, 296-8602 Chiba Japan; Hospital Pharmaceutics, Showa University Faculty of Pharmaceutical Sciences, 1-5-8 Hatanodai, Shinagawa-ku, 142-8666 Tokyo Japan

**Keywords:** Pravastatin sodium, Generic drugs, Long-term use

## Abstract

**Background:**

Increasing the use of generic drugs may reduce the growing healthcare spending. Nevertheless, in Japan, the generic drug market share remains low compared to that of European countries and the United States, mainly because of the general distrust of generic drugs. To address this problem, we retrospectively evaluated the efficacy and safety of the long-term use of generic pravastatin sodium in a study from January 2008 to December 2011.

**Methods:**

Patients receiving generic pravastatin sodium for ≥15 months were defined as long-term users and were included in the study, totaling 595 out of 1337 patients. Efficacy assessment was based on the total cholesterol (TC), triglyceride (TG), high-density lipoprotein (HDL), and low-density lipoprotein (LDL) plasma levels. Safety assessment was based on the aspartate aminotransferase (AST), alanine aminotransferase (ALT), creatine phosphokinase (CPK), gamma-glutamyl transferase (γ-GTP), alkaline phosphatase (ALP), lactate dehydrogenase (LDH), total-bilirubin (T-Bil), blood urea nitrogen (BUN), serum creatinine (Scr), and hemoglobin A1c (HbA1c) plasma levels. The patients’ reasons for discontinuing generic pravastatin sodium were obtained from the electronic medical records.

**Results & discussion:**

No significant difference in the laboratory data was observed between short-term and long-term users, except for significantly lower ALT levels in the long-term users than in the short-term users. No liver dysfunction was observed. Although 37 patients discontinued the study possibly owing to drug-related adverse events, we considered these events unrelated to generic pravastatin sodium.

**Conclusions:**

This study shows that the long-term use of generic pravastatin sodium is effective and safe, and may help dispel the concerns about generic drugs.

## Background

Increasing the use of generic instead of branded drugs is desirable since it contributes to reducing healthcare spending [1]. Although the generic drugs’ penetration rates are approximately 50 % or more in Europe and the United States, according to an aggregated value based on a drug price survey conducted in September 2013, they are 46.9 % in Japan, and are currently still low [2, 3]. To address this situation, the Japanese Ministry of Health, Labour and Welfare has set a goal to increase the volume share of generic drugs to 60 % or more by the fiscal year of 2018. Promotion of the use of generics also plays a large role in the medical economy [4].

One of the reasons for the persistently low penetration rate of generic drugs is the distrust of doctors and patients because less information on their efficacy and safety is available than that for branded drugs [5]. To address this issue, it is important not only to ensure the biological and therapeutic equivalence of generic drugs to those of branded drugs, but also to clinically assess their efficacy and safety.

When we switched from branded to generic pravastatin sodium tablets at our hospital, we assessed the clinical efficacy and safety of the generic version on the basis of laboratory data and reported our findings [6]. In this study, which included all 1337 patients who switched to the generic pravastatin sodium tablet Maibastan® (Towa Pharmaceutical, Co., Ltd.), we demonstrated that Maibastan®’s clinical efficacy and safety were comparable to those of the branded Mevalotin® tablet (Daiichi Sankyo Co., Ltd.). However, since the study period was 6 months and the long-term use of Mevalotin® was defined as 15 months or longer [6], the impact of Maibastan®’s long-term use on clinical efficacy and safety remains unknown. Moreover, although several clinical studies have compared and analyzed the efficacy and safety of branded and generic drugs in patients switching from branded to generic drugs, only few have investigated the clinical efficacy and safety of the long-term use of the latter [7–9]. Thus, in the present study, the duration of long-term use was defined as 15 months or longer [10], and the study period was extended to last from January 1, 2008 to December 31, 2011. All 1337 patients who had switched to Maibastan® in the previous study were followed up, and a retrospective observational study using information from electronic medical records to assess Maibastan®’s long-term use clinical efficacy and safety was conducted.

## Methods

### Study period

From January 1, 2008, to December 31, 2011.

### Design

Retrospective observational study.

### Target patients

**Fig. 1:**
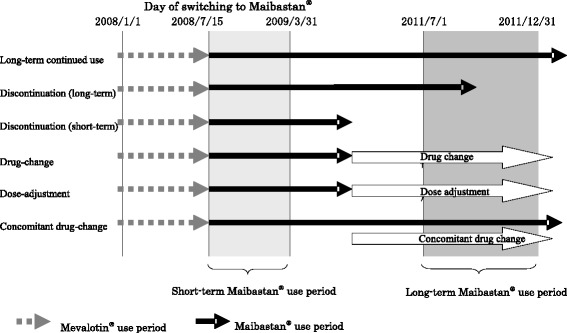
Overview of each treatment group and the study period. Short-term treatment period: From the day of substitution to March 31, 2009. Long-term treatment period: From July 1 to December 31, 2011. Long-term continued use group: Patients who continued the treatment on and after December 31, 2011. Treatment discontinuation group (long-term): Patients who discontinued the treatment between July 1 and December 31, 2011. Treatment discontinuation group (short-term): Patients who discontinued the treatment on or before July 1, 2011. Drug-change group: Patients for whom the generic drug was replaced by another drug after substitution. Dose-adjustment group: Patients for whom the dose of the generic drug was adjusted after substitution. Concomitant drug-change group: Patients whose concomitant drugs were switched after substitution. Long-term use group: A group consisting of the long-term continued use group and the treatment discontinuation group (long-term). Discontinuation/change group: A group consisting of the treatment discontinuation (short-term), drug-change, dose-adjustment, and concomitant drug-change group

The present study included all 1337 patients who had switched from branded pravastatin sodium (Mevalotin® tablet) to a generic version (Maibastan® tablet) between January 1, 2008, and March 31, 2009. These patients were classified into 6 different groups: long-term continued use group, treatment discontinuation group (long-term), treatment discontinuation group (short-term), drug-change group, dose-adjustment group, and concomitant drug-change group (Fig. 1). Definition of the long-term treatment period was defined as 15 months or longer of treatment according to a clinical study on the long-term use of Mevalotin® [10].

### Exclusion criteria

Patients for whom no efficacy and safety laboratory assessment data were obtained during the short- and long-term treatment periods were excluded from the study [5. Assessment items, 2)].

### Assessment items

1. The number of the target patients, male-to-female ratio, mean age, and mean duration of orally administered Maibastan® tablets
2. Efficacy and safety assessment based on laboratory data

- Efficacy assessment

**Fig. 2:**
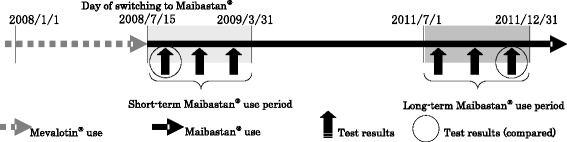
Comparison of laboratory data between the treatment groups

In the long-term use group, the results of the first laboratory test during the short-term treatment period (from the day of substitution to March 31, 2009) were compared with the results of the latest laboratory test during the long-term treatment period (from July 1 to December 31, 2011) (Fig. 2). The efficacy of the long-term use of Maibastan® tablets was assessed by comparing the following laboratory data: total cholesterol (TC), triglyceride (TG), high-density lipoprotein cholesterol (HDL-C), and low-density lipoprotein cholesterol (LDL-C).

- Safety assessment

In the long-term use group, the results of the first laboratory test during the short-term treatment period (from the day of substitution to March 31, 2009) were compared with those of the last laboratory test during the long-term treatment period (from July 1 to December 31, 2011) (Fig. 2). The safety of Maibastan® tablets’ long-term use was assessed by comparing the following laboratory data: aspartate aminotransferase (AST), alanine aminotransferase (ALT), creatine phosphokinase (CPK), gamma-glutamyl transferase (γ-GTP), alkaline phosphatase (ALP), lactate dehydrogenase (LDH), total bilirubin (T-Bil), blood urea nitrogen (BUN), serum creatinine (Scr), and hemoglobin A1c (HbA1c).

3) Safety assessment based on the reasons for drug discontinuation and changes

To assess the safety of Maibastan®, we reviewed the medical records to identify the reasons underlying all Maibastan® discontinuation and replacement cases and compared the cases of adverse reactions to Maibastan® with those reported for Mevalotin®. In the event of adverse reactions that had not been reported for Mevalotin® or of death, the patient data were reviewed to investigate a causal relationship with orally administered Maibastan®. The Common Terminology Criteria for Adverse Events version 4.0, Japan Clinical Oncology Group version (CTCAE, v4.0-JCOG), were used for grading of adverse reactions for the safety assessment.

### Analytical methods

Student’s *t-*test was used for intergroup comparison of the laboratory data. The statistical significance level was set at *p* < 0.05.

The study protocol was approved by the Clinical Study Screening Committee at Kameda General Hospital.

## Results

1. Sex and age of the target patients and treatment duration of orally administered Maibastan® tablets

Of the 1337 patients who had switched from Mevalotin® to Maibastan® tablets between January 1, 2008, and March 31, 2009, 595 patients continued receiving Maibastan® for approximately 177 weeks, whereas the remaining 742 patients discontinued or changed their treatment for undefined reasons. The long-term use group consisted of 556 and 39 patients in the long-term continued use and treatment discontinuation (long-term) group, respectively (Table 1). The discontinuation/change group consisted of 278, 329, 98, and 37 patients in the treatment discontinuation (short-term), drug-change, dose-adjustment, and concomitant drug-change group, respectively (Table 2). The efficacy and safety assessment based on laboratory data included the 595 patients in the long-term use group, while the safety assessment based on the reasons for discontinuation and drug change included 781 patients, i.e., 742 and 39 patients in the discontinuation/change and treatment discontinuation (long-term) group, respectively. No apparent difference was observed in the male-to-female ratio and mean age between the target patients.

**Table 1:** Patient characteristics. (1) Long-term use group

	Long-term continued use	Discontinuation (long-term)
Patients (N)	556	39
Men/women (N)	224/332	18/21
Mean age (years)	68.8 ± 10.9	73.8 ± 11.8
Mean treatment duration (weeks)	177 ± 3.61	158 ± 3.54

**Table 2:** Patient characteristics. (2) Discontinuation/change group

	Concomitant drug-change	Drug-change	Dose-adjustment	Discontinuation (short-term)
Patients (N)	37	329	98	278
Men/women (N)	24/13	150/179	44/54	103/175
Mean age (years)	66.7	66.8	68.1	70.9
Mean treatment duration (weeks)	144 ± 3.50	66.6 ± 3.53	143 ± 3.71	63.8 ± 4.00
	Age (years)		Duration (weeks)	
	Mean	S.D.	Mean	S.D.
Long-term continued use	68.8	10.9	177	3.61
Discontinuation (long-term)	73.8	11.8	158	3.54
Concomitant drug-change	66.7	11.0	144	3.50
Drug-change	66.8	11.1	66.6	3.53
Dose-adjustment	68.1	11.2	143	3.71
Discontinuation (short-term)	70.9	12.5	63.8	4.00

Standard deviation, S.D

2) Assessment of efficacy and safety based on laboratory data

- Efficacy assessment

In the long-term continued use group, no significant difference was observed in laboratory parameters between the Maibastan® tablets short- and long-term use groups (Table 3) and in those of the treatment discontinuation group (long-term) (Table 4).

**Table 3:** Assessment of efficacy and safety based on laboratory data (the long-term continual use group)

	Short-term treatment				Long-term treatment				
Plasma parameter	Mean ± S.D.			N	Mean ± S.D.			N	*p*-value
TC	195	±	25.0	407	196	±	28.5	417	0.42
TG	129	±	76.8	405	123	±	65.5	449	0.16
HDL-C	60.8	±	15.6	388	61.9	±	16.6	437	0.32
LDL-C	109	±	20.1	239	112	±	24.0	246	0.06
AST	24.2	±	12.2	419	23.2	±	9.29	473	0.19
ALT	23.1	±	21.6	418	20.4	±	11.3	473	0.02
CPK	112	±	86.5	358	108	±	67.9	395	0.51
γ-GTP	35.3	±	52.4	324	33.0	±	35.5	385	0.50
ALP	236	±	72.1	266	245	±	81.8	286	0.18
T-Bil	0.66	±	0.25	242	0.67	±	0.27	303	0.91
LDH	198	±	37.4	372	196	±	39.1	417	0.48
BUN	17.0	±	8.78	384	17.6	±	8.39	461	0.27
Scr	0.87	±	0.55	413	0.91	±	0.74	478	0.40
HbA1c	6.26	±	0.83	308	6.24	±	0.88	341	0.87

Total plasma cholesterol, TC; triglyceride, TG; high-density lipoprotein cholesterol, HDL-C; low-density lipoprotein cholesterol, LDL-C; aspartate aminotransferase, AST; alanine aminotransferase, ALT; creatine phosphokinase, CPK; gamma-glutamyl transferase, γ-GTP; alkaline phosphatase, ALP; lactate dehydrogenase, LDH; total bilirubin, T-Bil; blood urea nitrogen, BUN; serum creatinine, Scr; hemoglobin A1c, HbA1c; standard deviation, S.D

**Table 4:** Assessment of efficacy and safety based on laboratory data (the treatment discontinuation group)

	Short-term treatment				Long-term treatment				
Plasma parameter	Mean ± S.D.			N	Mean ± S.D.			N	*p*-value
TC	188	±	31.2	31	179	±	39.4	16	0.38
TG	117	±	51.1	30	109	±	50.0	17	0.62
HDL-C	61.4	±	22.6	28	59	±	18.5	17	0.71
LDL-C	102	±	19.2	20	96.6	±	26.4	11	0.56
AST	20.7	±	7.15	33	19.6	±	7.29	26	0.57
ALT	17.6	±	9.37	33	15.3	±	7.74	26	0.32
CPK	81.9	±	40.1	30	83.6	±	44.1	19	0.89
γ-GTP	31.7	±	37.4	26	39.1	±	42.9	21	0.53
ALP	237	±	69.3	23	285	±	134	21	0.16
T-Bil	0.76	±	0.48	24	0.57	±	0.25	20	0.10
LDH	190	±	35.1	30	182	±	56.0	22	0.54
BUN	16.6	±	5.46	35	19.3	±	9.33	27	0.19
Scr	0.85	±	0.28	35	0.91	±	0.41	27	0.52
HbA1c	6.32	±	0.95	22	6.15	±	0.4	11	0.47

Total plasma cholesterol, TC; triglyceride, TG; high-density lipoprotein cholesterol, HDL-C; low-density lipoprotein cholesterol, LDL-C; aspartate aminotransferase, AST; alanine aminotransferase, ALT; creatine phosphokinase, CPK; gamma-glutamyl transferase, γ-GTP; alkaline phosphatase, ALP; lactate dehydrogenase, LDH; total bilirubin, T-Bil; blood urea nitrogen, BUN; serum creatinine, Scr; hemoglobin A1c, HbA1c; standard deviation, S.D

- Safety assessment

Although in the long-term continued use group, a significant decrease in ALT levels (*p* = 0.02) was observed between the short- and long-term use of Maibastan® tablets, no significant difference was observed in any of the other laboratory parameters (Table 3). Moreover, no significant difference in laboratory parameters was observed in the treatment discontinuation group (long-term) (Table 4).

3) Safety assessment based on the reasons for drug discontinuation and change

Among the 39 patients in the treatment discontinuation group (long-term), the reasons for discontinuation were not clearly indicated in the medical records of 28 patients. In five patients, treatment was discontinued because of transfer to another hospital and because of death, pregnancy, poor nutritional status, and advanced age preventing chronic treatment in 3, 1, 1, and 1 patient(s), respectively.

Among the 278 patients in the treatment discontinuation group (short-term), the reasons for discontinuation were not clearly indicated in the medical records of 141 patients. In 62 patients, treatment was discontinued because of transfer to another hospital, and in 33, 21, 13, 3, 3, and 2 patients, because of death, adverse reactions, decreased LDL-C levels, poor nutritional status, patient’s own request, and old age preventing chronic treatment, respectively. Among the 21 patients who discontinued treatment because of adverse reactions, eight patients presented symptoms suggestive of rhabdomyolysis, such as increased CPK levels and myalgia.

Among the 329 patients in the drug-change group, the reasons for drug change were not clearly indicated in the medical records of 187 patients. Maibastan® was replaced by other drugs because of poor LDL-C control in 116 patients (Crestor® [AstraZeneca, Inc.; 61 patients], Lipitor® [Astellas Pharma, Inc.; 54 patients], and Bezatol® [Kissei Pharmaceutical Co., Ltd.; 1 patient]) and because of poor TG control in 9 patients (Crestor®, Lipitor®, Bezatol®, and Lipidil® [Kaken Pharmaceutical Co., Ltd.; 2 patients each] and Zetia® [Bayer AG; 1 patient]). Moreover, 1 patient switched from Maibastan® to another drug (Bezatol®) because of favorable LDL-C control. Adverse reactions caused all 16 patients to switch from the generic drug to other drugs (Lipitor® tablet in 9 patients, Crestor® and Zetia® tablet in 3 patients each, and Epadel® capsule [Mochida Pharmaceutical Co., Ltd.] in 1 patient). Nine of the patients presented symptoms suggestive of rhabdomyolysis, such as increased CPK levels, myalgia, and weakness.

Among the 98 patients in the dose-adjustment group, the reasons for drug change were not clearly indicated in the medical records of 33 patients. In 37 patients, the doses of Maibastan® tablet were reduced because of favorable LDL-C control, and in 27 and 1 patient(s), because of poor LDL-C and TG control, respectively.

Among the 37 patients in the concomitant drug-change group, the reasons for drug change were not clearly indicated in the medical records of 17 patients. In 11 patients, other drugs were added as treatment because of poor LDL-C control (Zetia® tablet in 10 patients and Bezatol® tablet in 1 patient) and poor TG control in 9 patients (Zetia® and Bezatol® tablet in 3 patients each, Lipidil® tablet in 2 patients, and Perycit® tablet [Sanwa Kagaku Kenkyusho Co., Ltd.] in 1 patient).

During the current study period, treatment was discontinued or changed because of adverse reactions in a total of 37 patients. Among them, abnormal laboratory data were observed in 13 patients, and 24 patients reported adverse reactions. The adverse reactions determined by abnormal laboratory data in the 13 patients were increased CPK, AST and ALT, ALP and γ-GTP and decreased Hb levels in 9, 1, 1, and 1 patient(s), respectively. Thrombocytopenia was observed in 1 patient (Table 5).

**Table 5:** Reports of adverse reactions determined by abnormal laboratory data

Age (years)	Sex	Treatment duration (weeks)	Adverse reaction			CTCAE Grade
70	Female	16	Increased CPK	200	IU/L	I
66	Female	10	Increased CPK	219	IU/L	I
67	Female	40	Increased CPK	189	IU/L	I
68	Female	15	Increased CPK	200	IU/L	I
78	Female	4	Increased CPK	2898	IU/L	IV
52	Male	12	Increased CPK	321	IU/L	I
67	Male	35	Increased CPK	762	IU/L	I
36	Male	4	Increased CPK	404	IU/L	I
40	Male	10	Increased CPK	815	IU/L	II
69	Female	4	Increased AST	49	IU/L	I
41	Male	32	Increased ALT	42	IU/L	I
			Increased ALP	376	IU/L	I
			Increased γ-GTP	194	IU/L	II
65	Male	23	Decreased Hb	9.7	g/dL	-
85	Female	15	Thrombocytopenia	65,000	/mL	II

Aspartate aminotransferase, AST; alanine aminotransferase, ALT; creatine phosphokinase, CPK; gamma-glutamyl transferase, γGTP; alkaline phosphatase, ALP; hemoglobin, Hb; The Common Terminology Criteria for Adverse Events, CTCAE

Moreover, CTCAE grade II or higher adverse reactions were observed in 2 patients with increased CPK levels (grade II and IV) and in 1 patient each with increased γ-GTP levels, decreased Hb levels, and thrombocytopenia (all were grade II). The adverse reactions reported by 24 patients were myalgia in 7, rash in 6, discomfort in 3, and myospasm in 2 patients, as well as arrhythmia, numbness, malaise, nausea, weakness, and vertigo in 1 patient each. Of these reported adverse reactions, muscle-associated adverse reactions suggestive of rhabdomyolysis (increased CPK levels, myalgia, myospasm, numbness, malaise, and weakness) accounted for a large proportion of the patients (21 out of 37).

Comparison with the reported adverse reactions to orally administered Mevalotin® revealed no apparent increase in the incidence of adverse reactions to orally administered Maibastan® tablets (Table 6). Moreover, 3 cases of discomfort and 1 case of arrhythmia, which were not reported as adverse reactions to Mevalotin®, were examined in detail using the information in the electronic medical records (e.g., past history and concomitant drugs).

**Table 6:** Comparison of adverse reactions reported for the branded and generic drugs

Adverse reaction	Case (N)	Present study	Package insert
		Incidence (%)	Incidence (%)
Increased CPK	9	0.67	Unknown
Myalgia	7	0.52	Unknown
Rash	6	0.45	0.1-1
Discomfort	3	0.22	No documentation
Myospasm	2	0.15	<0.1
Arrhythmia	1	0.07	No documentation
Increased ALP and γ-GTP	1	0.07	0.1–1
Numbness	1	0.07	<0.1
Malaise	1	0.07	<0.1
Nausea	1	0.07	<0.1
Increased AST and ALT	1	0.07	Unknown
Thrombocytopenia	1	0.07	Unknown
Anemia (decreased Hb)	1	0.07	Unknown
Weakness	1	0.07	Unknown
Vertigo	1	0.07	Unknown

Creatine phosphokinase, CPK; gamma-glutamyl transferase, γ-GTP; alkaline phosphatase, ALP; aspartate aminotransferase, AST; alanine aminotransferase, ALT; hemoglobin, Hb

Case 1 is a 48-year-old man treated for dyslipidemia on an outpatient basis. Ten months after switching to Maibastan® tablet, he complained of discomfort, and drug treatment was discontinued. The patient’s discomfort was related to the use of Maibastan® tablet together with 2 drugs prescribed for sinusitis at another hospital (unspecified). The other concomitant drugs used were Bayaspirin® tablet (Bayer AG), Juvela® capsule (Eisai Co., Ltd.), and Foliamin® tablet (Takeda Pharmaceutical Co., Ltd.). Discontinuation of all drugs relieved the patient’s discomfort.

Case 2 is a 76-year-old man treated for chronic renal failure, renal anemia, hypertension, prostatomegaly, and dyslipidemia on an outpatient basis. Two months after switching to Maibastan® tablet, he complained of discomfort, and drug treatment was discontinued. Because of discomfort, the patient discontinued the use of Urief® (Daiichi Sankyo Co. Ltd.), Maibastan®, Tenormin® (AstraZeneca, Inc.), and Myslee® tablet (AstraZeneca, Inc.) at his own discretion. The patient’s discomfort was subsequently relieved. The use of the other concomitant drugs, Adalat® (Bayer AG) and Blopress® tablet (Takeda Pharmaceutical Co., Ltd.), was not discontinued.

Case 3 is an 88-year-old woman treated for hypothyroidism and dyslipidemia on an outpatient basis. Two months after switching to Maibastan® tablet, she complained of discomfort, and drug treatment was discontinued. Because of discomfort, the patient discontinued Maibastan® and Hachimi-jio-gan® extract tablet (Kracie Pharma, Ltd.) at her own discretion. The patient’s discomfort was subsequently relieved. The use of the other concomitant drug, Thyradin-S® tablet (Takeda Pharmaceutical Co. Ltd.), was not discontinued.

Case 4 is a 66-year-old woman treated for diabetes mellitus, hypertension, and dyslipidemia on an outpatient basis. Twenty months after switching to Maibastan® tablet, she complained of a racing pulse, and treatment was discontinued. This patient discontinued Maibastan® tablet at her own discretion and her symptom was subsequently relieved. The use of the concomitant drugs Olmetec® (Daiichi Sankyo Co., Ltd.) and Fastic® tablet (Mochida Pharmaceutical Co., Ltd.) was not discontinued (Table 7).

**Table 7:** Cases of adverse reactions that have not been reported for the branded drug

Adverse reaction	Course
Discomfort	A 48-year-old man complained of discomfort after receiving Maibastan® for 10 months, and the treatment was discontinued. Before symptom onset, the concomitant drugs had not been changed. Discontinuation of Maibastan® and the concomitant drugs* resulted in symptom relief.
	*Bayaspirin®, Juvela®, Foliamin®, Erythrocin®, and Mucodyne®
	A 76-year-old man complained of discomfort after receiving Maibastan® for 2 months, and the treatment was discontinued. Before symptom onset, the concomitant drugs* had not been changed. Discontinuation of Maibastan®, Urief®, Tenormin®, and Myslee® resulted in symptom relief.
	*Urief®, Tenormin®, Myslee®, Adalat®, and Blopress®
	An 88-year-old woman complained of discomfort after receiving Maibastan® for 2 months, and drug treatment was changed to Epadel®. Before symptom onset, the concomitant drugs* had not been changed. Discontinuation of Maibastan® and Hachimi-jio-gan® resulted in symptom relief.
	*Thyradin S® and Hachimi-jio-gan®
Arrhythmia	A 66-year-old woman complained of the sensation of having a racing pulse after receiving Maibastan® for 20 months, and the treatment was discontinued. Before symptom onset, the concomitant drugs* had not been changed. Discontinuation of Maibastan® resulted in symptom relief.
	*Olmetec® and Fastic®

## Discussion

Efficacy and safety assessment based on laboratory data included those of the patients in the long-term use group, which consisted of 556 patients in the long-term continued use and 39 patients in the treatment discontinuation (long-term) group. Regarding the efficacy in the long-term continued use group, no statistically significant difference was observed between the first TC, TG, HDL-C, and LDL-C levels measured during the short-term use of Maibastan® tablets and those of the last measured during the long-term treatment period. Furthermore, no significant difference between these parameters was observed in the treatment discontinuation group (long-term). Thus, it was assumed that the effects of the long-term use of Maibastan® tablets were similar to those of Mevalotin® tablets.

Similarly, regarding the safety assessment in the long-term continued use group, no statistically significant difference was observed between the first AST, ALT, CPK, γ-GTP, ALP, LDH, T-Bil, BUN, Scr, and HbA1c levels measured during the short-term use of Maibastan® tablet period and those of the last measurement during the long-term treatment period, except for the ALT levels. While the ALT levels were significantly lower in the Maibastan® tablet long-term use group than those in the short-term use group, the levels did not fluctuate throughout the long-term treatment period. A recent report from the Greek Atorvastatin and Coronary Heart Disease Evaluation (GREACE) study indicated that the use of statins in patients with a fatty liver improved liver function and reduced cardiovascular events [11]. As for the reason for the decreased ALT levels, the report also suggested that the inhibitory action of Maibastan® against hepatic 3-methylglutaryl coenzyme A (HMG-CoA) reductase reduced stress, such as a fatty liver, affecting liver function. A similar comparison of the laboratory parameter values in the treatment discontinuation group showed no significant differences.

For the safety assessment, review of the patients’ medical records identified the reasons underlying all cases of drug discontinuation and change during the current study period. Detailed review of the medical records containing no documented reasons revealed the absence of patients’ complaints about symptoms suggestive of adverse reactions, abnormal laboratory data during treatment, or adverse events, such as death.

Next, to investigate the safety of the long-term use of Maibastan® tablets, the medical records of patients who had died or had adverse reactions were reviewed in detail. Among all the 36 patients who had died, drug discontinuation and change were suspected to be caused by primary diseases, and it was assumed that there was no causal relationship with the oral administration of Maibastan® tablets. In the drug discontinuation and change due to adverse reactions cases, Maibastan® tablet was considered responsible for each adverse reaction. Indeed, 21 out of the 37 cases showed muscle-associated symptoms suggestive of rhabdomyolysis (increased CPK levels, myalgia, myospasm, numbness, malaise, and weakness). However, these findings were derived from pravastatin sodium preparations and may be considered Maibastan® tablet-unrelated adverse reactions. Moreover, the adverse reactions observed in 12 out of the 37 cases, which included rash, increased ALP and γ-GTP levels, nausea, hepatopathy, thrombocytopenia, decreased Hb levels, and vertigo, were also reported for the branded drug (Mevalotin®), and no apparent increase in their incidence was observed. Thus, these adverse reactions may also be considered Maibastan® tablet-unrelated. Because discomfort (case 1, 2, and 3) and arrhythmia (case 4), which were observed in the remaining 4 cases, had not been reported in the adverse reaction reports for the branded drug, we investigated whether these adverse reactions were specific to Maibastan® tablet treatment. Although discomfort was relieved in these 3 cases(case 1, 2, and 3) after discontinuation of Maibastan® tablet, this adverse reaction may not be specific to the tablet: the patients were taking several oral drugs and had comorbidities associated with poor physical conditions, such as sinusitis, renal anemia, and hypothyroidism. Although the symptom(case 4) was relieved after discontinuation of Maibastan® tablet only, it has been reported that arrhythmic symptoms, such as extrasystole, are detected in approximately 3.8 % of healthy people, and that the incidence of such symptoms is higher in patients with hypertension [12, 13]. Because the patient of case 4 had concomitant hypertension, it cannot be ruled out that the racing pulse she experienced was caused by transient extrasystole. Thus, this symptom may not be a Maibastan® tablet-specific adverse reaction. According to the findings described above, it is assumed that Maibastan® tablet may be safely used for long-term treatment.

We previously conducted questionnaire surveys on generic drugs among doctors, nurses, and pharmacists at our hospital and found that several healthcare professionals had concerns about the limited amount of information on clinical efficacy and adverse reactions [14, 15]. Although several studies have examined the efficacy and safety of generic drugs, the number of studies focusing on individual drugs and the amount of information provided by such studies has not been sufficient to eliminate this concern. Some studies have also assessed the efficacy and safety of generic pravastatin sodium tablets. However, in several of these studies, less than 100 cases were evaluated, and the duration of generic drug treatment lasted for 3 months or less; even the longest duration lasted 6 months at most [16–19]. Drugs for the treatment of chronic diseases are often orally administered for many years. Especially, for dyslipidemia, strict long-term lipid control is necessary for the primary and secondary prevention of cardiovascular events. Thus, studies on the efficacy and safety of long-term drug use are highly useful information sources. The present study provides results on the efficacy and safety of a generic drug based on the assessment of a sufficient number of cases and sufficient treatment duration. The proliferation of generic drugs is highly desired to reduce healthcare spending. However, the major future tasks are not only to ensure their bioequivalence with branded drugs, but also to increase data provision on generic drug efficacy and safety by conducting clinical studies. In these tasks, pharmacists are essential since they are drug specialists. Therefore, pharmacists should be actively involved through pharmacist-led clinical assessments of generic drugs. The present study may have large clinical significance for the efficacy and safety assessment of the long-term use of generic drugs.

## Conclusion

This study shows that the long-term use of generic pravastatin sodium is effective and safe, and may help dispel the concerns about generic drugs.
